# FBXW7 metabolic reprogramming inhibits the development of colon cancer by down-regulating the activity of arginine/mToR pathways

**DOI:** 10.1371/journal.pone.0317294

**Published:** 2025-01-17

**Authors:** Qing Li, Yan Li, Tong Zhou, Yong Zhang, Huiyu Li, Fajia Yuan, Yanghui Bi

**Affiliations:** 1 Center of Gene Sequencing, Shanxi Bethune Hospital, Shanxi Academy of Medical Sciences, Tongji Shanxi Hospital, Third Hospital of Shanxi Medical University, Taiyuan, P. R. China; 2 Department of Epidemiology, Academy of Medical Sciences, School of Public Health, Shanxi Medical University, Taiyuan, P. R. China; 3 Academy of Medical Sciences, Shanxi Medical University, Taiyuan, China; 4 Endoscopic Center of Shanxi Province Cancer Hospital, Shanxi Hospital Affiliated to Cancer Hospital, Chinese Academy of Medical Sciences, Cancer Hospital Affiliated to Shanxi Medical University, Taiyuan, P. R. China; 5 Department of General Surgery, Shanxi Bethune Hospital, Shanxi Academy of Medical Sciences, Tongji Shanxi Hospital, Third Hospital of Shanxi Medical University, Taiyuan, China; 6 Shanxi Jinzhong Health School, Jinzhong, P. R. China; Sun Yat-Sen University, CHINA

## Abstract

FBXW7 is a tumor suppressor gene that regulates metabolism and is associated with the onset and progression of colorectal cancer (CRC)), however, the precise mechanism whereby FBXW7 participates in the metabolic reprogramming of CRC remains unclear. Here, the research aims to reveal the association between the expression of FBXW7 and clinical variables and to investigate the molecular mechanism by which FBXW7 plays a critical role in the development of CRC. The clinical importance of FBXW7 in CRC was determined by immunohistochemistry. Non-targeted metabolomics was utilized to explore the role of FBXW7 in the metabolic regulation of CRC. Low expression of FBXW7 was associated with poor prognosis in individuals with CRC, both at the mRNA and protein levels. FBXW7 over-expression inhibited CRC cell growth, colony formation, migration, and invasion. Non-targeted metabolomics unveiled that FBXW7 over-expression directly caused the deprivation of arginine which led to downmodulation of mTOR signaling pathway; meanwhile, FBXW7-related metabolites were primarily concentrated in the mTOR signaling pathway. In summary, the research identified a novel mechanism of action of FBXW7 in CRC. The research findings provide a theoretical foundation for the prognostic prediction and therapeutic planning of CRC based on metabolic reprogramming.

## Introduction

Colorectal cancer (CRC) is the third most prevalent malignant tumor and the second leading cause of cancer-related mortality [1,2]. China has the highest number of new cases and fatalities from CRC in the world and is distinguished by a younger age of commencement, a higher degree of malignancy, and greater resistance to medication [3,4]. At present, CRC treatment is primarily focused on surgery along with radiation, chemotherapy, and targeted therapy; however, for patients who are not diagnosed early, there is typically little hope of success, even when resorting to drastic surgery. Simultaneously, with a 5-year survival rate of only 15%, the complete treatment shows insufficient efficacy in patients with recurrence and metastasis [1,5]. The FDA has authorized various targeted medications for CRC in recent decades; however, only 20% of patients benefit from them [6]. Most patients with advanced CRC have no medications available for backup treatment, and monitoring the metastasis and recurrence is still based on pan-tumor markers (CEA, CA124, and CA199) with low specificity and sensitivity [7,8]. Therefore, it is necessary to investigate the pathophysiology of the high-incidence CRC in China and identify new diagnostic and therapeutic targets to improve preventive and treatment outcomes.

Metabolic reprogramming is a hallmark of malignant tumors [9–11]. The activation of oncogenes and/or inactivation of tumor suppressor genes changes the metabolism of cancer cells, encouraging tumor development and progression [12–14]. Post-translational protein modifications regulate signaling pathways associated with cancer metabolism [15–18], and ubiquitination is the most prevalent post-translational protein modification [19–21]. For example, AKT-PI3K-mTOR signaling pathway-related proteins can be ubiquitinated by multiple E3 ligases, thus regulating glucose and lipid metabolism in tumors [22]. Recent studies have also implicated aberrant expression and activity of E3 ligase in the molecular etiology and pathophysiology of cancer incidence and progression [23]. Therefore, understanding the involvement of E3 ligases is key to revealing the mechanisms of metabolic reprogramming in cancer. FBXW7 is located on chromosome 4q31q.3 and encodes SKP1-CUL1-F-box-protein (SCF) E3 ubiquitin ligase, a member of the F-box protein family [24]. It targets ubiquitination to destroy several oncogenes (including c-Myc, Notch1, Notch4, c-Jun, and cyclin E) and takes part in many crucial biological processes, including metabolic reprogramming, DNA damage repair, cell cycle, immune response, and cell death [25]. However, data on FBXW7 metabolic regulation are far from complete.

In this study, a tissue microarray containing 51 pairs of CRC and normal tissues was used to reveal the association between the expression of FBXW7 and clinical variables via immunohistochemistry (IHC). Through non-targeted metabolomic detection in FBXW7-overexpressing CRC cell lines, the molecular mechanism by which FBXW7 plays a critical role in the development of CRC was explored. The low FBXW7 expression was found to be related to poor prognosis in patients with CRC and that FBXW7 functions as a tumor suppressor gene in CRC cells, inhibiting the development of CRC via the mechanism: reduction in arginine production through metabolic reprogramming and direct down-regulation of mToR signaling. Most notably, metabolic enzymes were identified in tumor samples from 20 patients with CRC. Using data from The Cancer Genome Atlas (TCGA) Program database, the relationship between FBXW7 and CRC-related metabolic genes was verified. The research findings indicate a putative mechanism by which FBXW7 loss-of-function contributes to the progression of CRC, implying a potential therapeutic target for people with low expression of FBXW7.

## Materials and methods

### Cell culture and transfection

The colorectal cell lines Lovo and HCT116 were stored at General Surgery Department of Shanxi Bethune Hospital. Cells were grown in DMEM high glucose media containing 10% FBS, 100U/ml penicillin, and 100g/ml streptomycin. Short Tandem Repeats (STR) was used to identify all cell lines that were free of mycoplasma infection.

The full-length sequence of FBXW7 gene was constructed using pCDNA3 vector. Liposome 2000 (Invitrogen, USA) was used for plasmid transfection, according to the product instructions. The transfection method was introduced in our previous study [26].

### Western blot

The cells were cultured for 4 days in the presence of either 0, 50 or 100 μM Arg. The cell precipitation was treated with a suitable amount of radio immunoprecipitation assay (RIPA) buffer including protease and phosphatase inhibitors, and the total protein was extracted by cold incubation. The protein concentration was determined using the BCA kit. The protein was transferred to a Polyvinylidene Difluoride (PVDF) membrane after separation on a 10% SDS-polyacrylamide gel. After blocking for 2 hours at room temperature with 5% skim milk, the protein was incubated with particular antibody overnight at 4°C. The next day, the second antibody was incubated at room temperature for 1 hour, and GelView was utilized to detect protein imprinting. Western blotting using FBXW7 antibody was used to evaluate the protein level in colorectal cell lines, and GAPDH was employed as the internal reference protein.

### qPCR

The quantitative real-time PCR (qRT-PCR) analysis was performed as previously described. The method was described in a previous study [26]. Total RNA was extracted from cells and reverse transcribed into cDNA. All qPCR experiments included a no template control and were done in triplicate and repeated three times at least. Specific primer sequences were used for qPCR. The primer sequences used in this study are listed in Table in S1 Table.

### MTT assay

The single cell solution was injected in a 96 well plate at a density of 4×10^3^/well to detect changes in the viability of CRC cells. At 24h, 48h, 72h, and 96h, 20μl 5mg/ml Methylthiazolyldiphenyl-tetrazolium bromide (MTT) (Invitrogen) was added to each well for 4 hours before discarding the mixture. At room temperature, 150μl Dimethyl sulfoxide (DMSO) was applied to each well for 15 minutes. An enzyme labeled equipment was used to detect absorbance at 490nm. Each experiment had five replicates, as well as at least three distinct experiments.

### Cell migration and invasion assay

To detect cell migration and invasion, we employed a Biocoat Matrigel invasion chamber. With 5×10^4^ cells/well, a single cell suspension without FBS was injected in the top chamber of a 24-well plate, and full media was added to the lower chamber. The top chamber was removed after 24 hours and treated with 4% paraformaldehyde. The migrating cells to the sub-membrane surface were examined using an Olympus microscope (Japan) after 0.1% crystal violet staining. Four visual areas were chosen at random for manual counting. Before beginning the invasion experiment, cover the membrane surface with Matrigel (BD bioscience). Each experiment had three holes, and at least three separate experiments were carried out.

### Colony formation assay

In 6-well plates with full DMEM high glucose media, cells were planted at 500 cells per well and allowed to develop for 2 weeks. Cells were fixed with 4% paraformaldehyde and stained with 1% crystal violet on day 14 before being manually counted. The experiment was performed in triplicate and repeated three times at least.

### Flow cytometry apoptosis detection

Flow cytometry apoptosis detection. CRC cells were detached with EDTA-free trypsin, centrifuged to collect cell pellet, washed with PBS, resuspended with 500 μl binding buffer, mixed with 5 μl of Annexin V-FITC (Solarbio life Science, China), and 10 μl of Propidium Iodide (Solarbio Life Science, China). After incubation in the dark at room temperature for 20 min, the cells were detected by flow cytometry (Beckman Coulter Life Science, USA). We did this experiment at least three times.

### Immunohistochemistry

Shanghai Xinchao Biological Co., Ltd. provided Tissue Microarrays (TMA) (HcolA180Su14). To detect FBXW7, immunohistochemistry (IHC) was used. Briefly, tissues were fixed in 4% paraformaldehyde and embedded in paraffin. Sections were treated with a particular antibody (Abcam) at an optimal dilution (1:200) overnight at 4°C. The slides were then stained with hematoxylin and stained with PV8000 (Zhongshan) and the Diaminobenzidine (DAB) detection kit (Maixin). All pictures were taken at Axio scan z1. The quantity of the protein of interest was determined using the Aperio Nuclear v.9 program. SPSS 19.0 was used for statistical analysis.

### Sample preparation

The cell precipitation was extracted in 50 μL of 15% phosphoric acid with 10 μL of 75 μg/mL 4-methylvaleric acid solution as IS and 140 μL ether. Subsequently, the samples were centrifuged at 4°C for 10 min at 12000 rpm after vortexing for 1 min and the supernatant was transferred into the vial prior to LC-MS analysis.

### LC-MS analysis

The LC analysis was performed on a ACQUITY UPLC System (Waters, Milford, MA, USA). Chromatography was carried out with an ACQUITY UPLC ® HSS T3 (150×2.1 mm, 1.8 μm) (Waters, Milford, MA, USA). The column maintained at 40°C. The flow rate and injection volume were set at 0.25 mL/min and 2 μL, respectively. For LC-ESI (+)-MS analysis, the mobile phases consisted of (C) 0.1% formic acid in acetonitrile (v/v) and (D) 0.1% formic acid in water (v/v). Separation was conducted under the following gradient: 0~1 min, 2% C; 1~9 min, 2%~50% C; 9~12 min, 50%~98% C; 12~13.5 min, 98% C; 13.5~14 min, 98%~2% C; 14~20 min, 2% C. For LC-ESI (-)-MS analysis, the analytes were carried out with (A) acetonitrile and (B) ammonium formate (5mM). Separation was conducted under the following gradient: 0~1 min, 2% A; 1~9 min, 2%~50% A; 9~12 min, 50%~98% A; 12~13.5 min, 98% A; 13.5~14 min, 98%~2% A; 14~17 min, 2% A.

Mass spectrometric detection of metabolites was performed on Q Exactive (Thermo Fisher Scientific, USA) with ESI ion source. Simultaneous MS1 and MS/MS (Full MS-ddMS2 mode, data-dependent MS/MS) acquisition was used. The parameters were as follows: sheath gas pressure, 30 arb; aux gas flow, 10 arb; spray voltage, 3.50 kV and -2.50 kV for ESI(+) and ESI(-), respectively; capillary temperature, 325°C; MS1 range, m/z 81–1000; MS1 resolving power, 70000 FWHM; number of data dependant scans per cycle, 10; MS/MS resolving power, 17500 FWHM; normalized collision energy, 30%; dynamic exclusion time, automatic.

### Statistical analysis

According to receiver operating characteristic (ROCs) curves, all samples and subgroup samples were classified as FBXW7_high_ or FBXW7_low_. Rank sum and chi-squared (*χ*^2^) tests were used to examine differences in the expression levels of FBXW7 in colorectal tissues with other clinic-pathological characteristics. To evaluate the cumulative survival time across various FBXW7 groups with clinic-pathological variables, Kaplan-Meier estimator and the log-rank test were performed. A Cox proportional hazards regression model was utilized to perform univariate and multivariate survival analyses. All tests were conducted at least three times, and the results were expressed as mean ± SD. For statistical examination of the data comparing the two groups, Student’s *t*-test was utilized. For the data above the two groups, one-way analysis of variance (ANOVA) and Dunnett’s test were performed. Spearman correlation analysis was performed in GraphPad Prism 7 software to elucidate the relationship between FBXW7 and the expression of other genes. The SPSS 19.0 statistical software program (IBS SPSS, Armonk, NY, USA) was employed to process all data. A *p* value of less than 0.05 was considered statistically significant.

## Results

### Low expression of FBXW7 protein is associated with poor prognosis in patients with CRC

**Fig 1:**
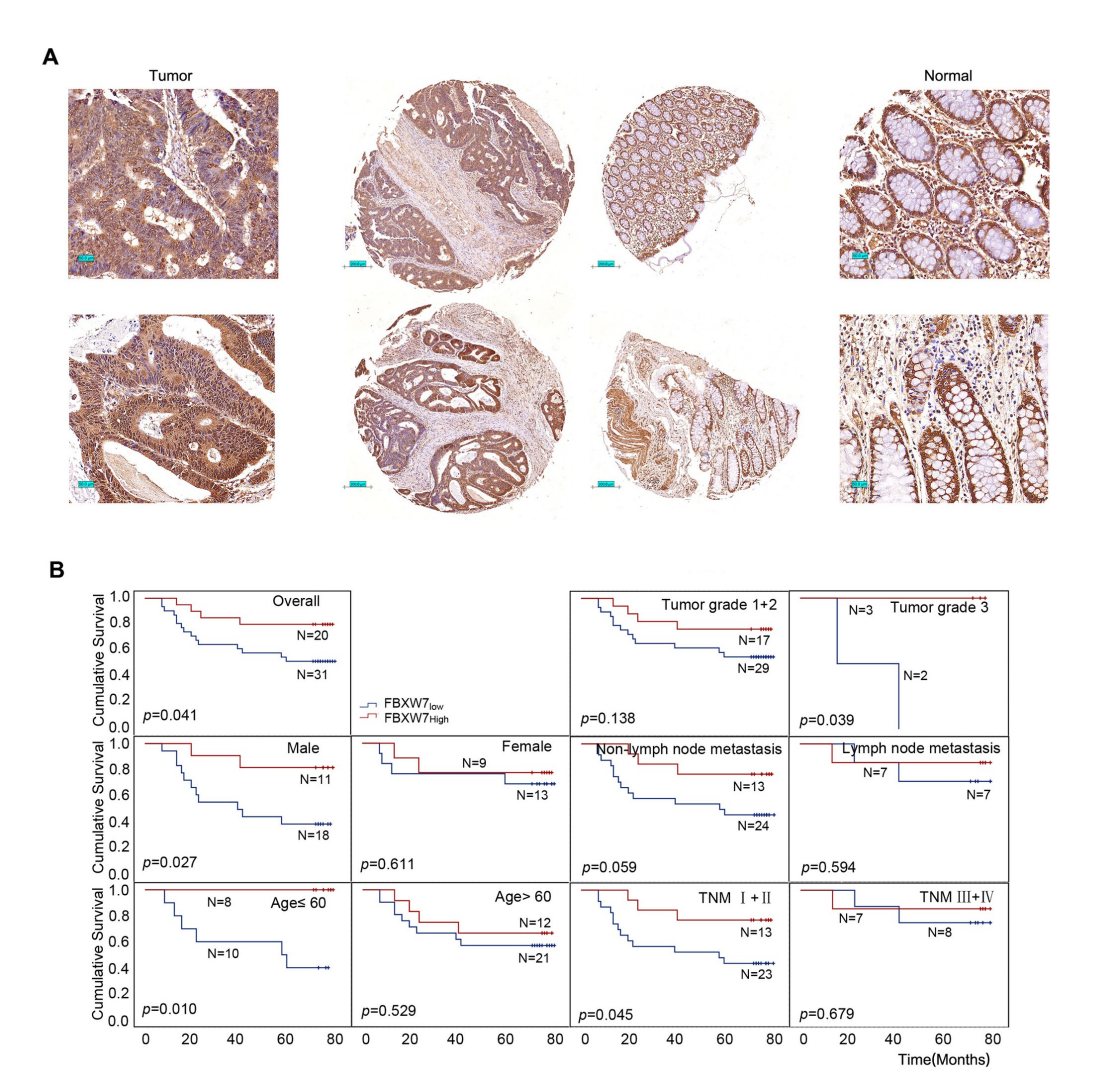
Prognostic values of CRC patients with different expression levels of FBXW7 protein. (A) Representative immunohistochemistry images of FBXW7 expression in tumor tissues and normal tissues from paraffin-embedded formalin-fixed CRC tissue microarrays including 51 tumors and corresponding non-tumor tissues. Scale bars represent 200 μm. (B) Kaplan-Meier survival curves of patients with different expression levels of FBXW7 in overall population and in patients with different genders, ages, tumor grades, lymph node metastases, and TNM stages.

IHC was utilized to determine the expression of FBXW7 protein in 51 CRC and matched normal tissues using a tissue microarray. The findings revealed that FBXW7 protein was localized in the nucleus and cytoplasm of CRC and normal tissues (**Fig 1A**). FBXW7 has three subtypes (FBXW7α, FBXW7β, and FBXW7γ) that are found in the nucleus, cytoplasm, and nucleolus, respectively. The most expressed subtype, FBXW7α, inhibits tumors via ubiquitination of oncogenic proteins. Therefore, the ratio of FBXW7 nuclear to cytoplasmic expression in tumor tissues was analyzed based on the ROC curve to elucidate the association between FBXW7 protein levels and ANOVA in CRC, and the optimal critical value of 1.875 with the maximal Youden index was determined. All CRC cases were divided into two groups based on FBXW7 levels: FBXW7_low_ (≤ 1.875) and FBXW7_high_ (> 1.875). The OS for FBXW7_low_ patients ranged from 7 to 78 months (median, 69 months). The FBXW7_high_ OS ranged from 13 to 77 months, with a median of 73 months. The rank-sum test indicated that patients in the FBXW7_low_ group had a shorter total cumulative survival time than those in the FBXW7_high_ group (*p* = 0.029). Kaplan-Meier survival analysis also suggested that the OS of the FBXW7_high_ group was significantly longer than that of the FBXW7_low_ group (*p* = 0.041), and the cumulative survival time of FBXW7_high_ patients was significantly longer than that of FBXW7_low_ patients in male (*p* = 0.027), aged 60 years old (*p* = 0.010), at pathological grade 3 (*p* = 0.039), and with TNM stage I + II (*p* = 0.045) (**Fig 1B**). Furthermore, the research discovered that while there was no significant difference in the cumulative survival time between FBXW7_high_ and FBXW7_low_ patients without lymph node metastases, FBXW7_high_ patients had a longer cumulative survival time than FBXW7_low_ patients.

### Low expression of FBXW7 mRNA is associated with poor prognosis in patients with CRC

**Fig 2:**
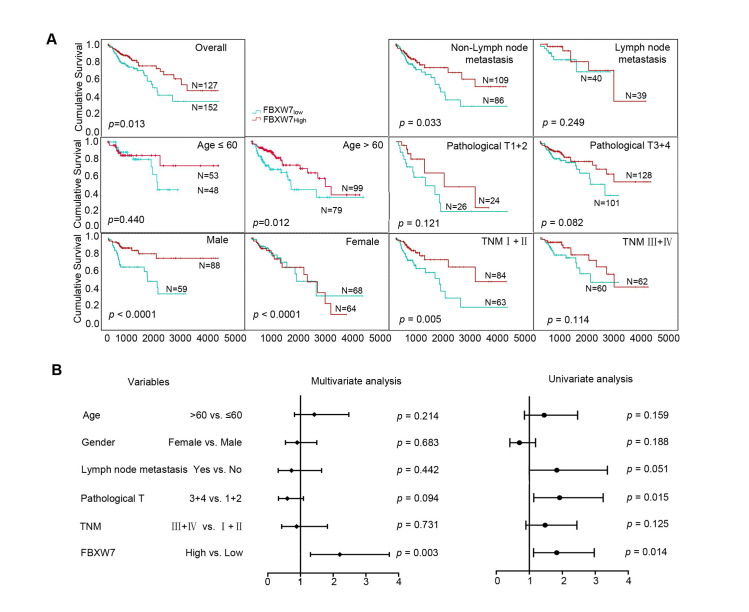
Prognostic values of CRC patients with different FBXW7 mRNA expression levels. (A) Kaplan-Meier survival curves of patients with different FBXW7 expression levels in overall population and in patients with different genders, ages, pathology T, tumor grades, lymph node metastases, and TNM stages. (B) Multivariate and univariate analysis by the Cox proportional hazards regression model in overall population.

In addition, TCGA data for 279 patients with CRC were examined and the mRNA expression level of FBXW7 was found to be related to age, sex, TNM stage, T pathology, and lymph node metastases. According to ROC curve analysis, samples were split into two groups: FBXW7_low_ (≤ 0.42985) and FBXW7_high_ (≥ 0.42985). Kaplan-Meier survival analysis revealed that FBXW7_low_ patients had a shorter survival time than FBXW7_high_ patients. The OS showed statistically significant relationships with the age, sex, lymph node metastasis, and TNM stage. In particular, the OS of FBXW7_low_ patients was shorter than that of FBXW7_high_ patients in CRC samples from patients aged > 60 years, of male sex, without lymph node metastases, and with TNM stage I + II (**Fig 2A**).

The Cox proportional hazards regression model for univariate and multivariate analyses was adopted to assess the predictive value of FBXW7 mRNA levels for cumulative survival of patients with CRC. Univariate analysis indicated that T pathology (HR, 1.92; 95% CI, 1.136–3.238; *p* = 0.015) and low FBXW7 expression (HR, 1.83; 95% CI, 1.129–2.969; *p* = 0.014) were significant risk factors for OS. Further multivariate analysis confirmed that FBXW7 was an independent predictor for predicting the overall cumulative survival status (HR, 2.202; 95% CI, 1.305–3.716; *p* = 0.003) (**Fig 2B**).

### Overexpression of FBXW7 inhibits the proliferation, invasion, and migration of CRC cells

**Fig 3:**
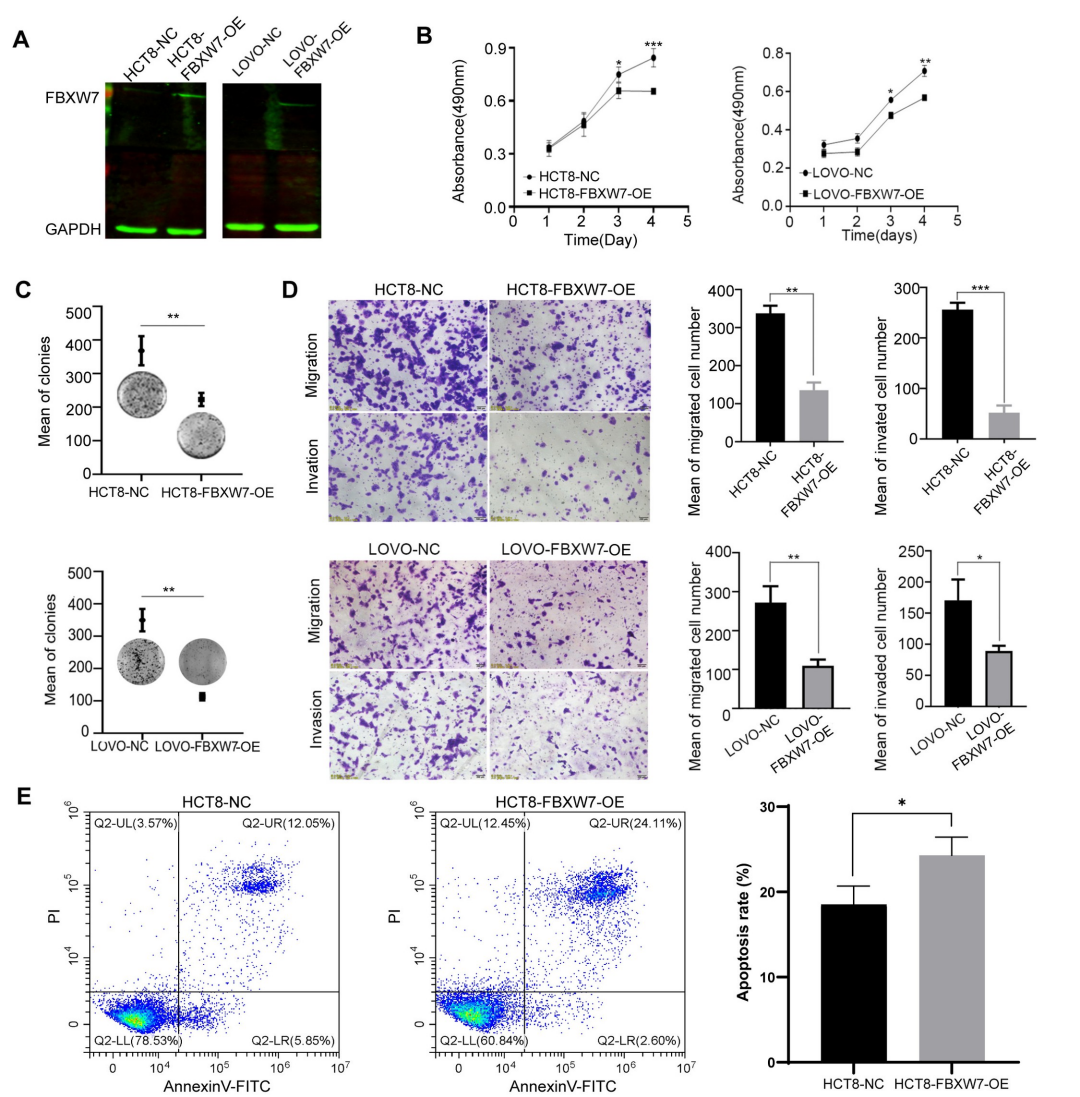
FBXW7 acts as a depression-gene affecting CRC cell growth, colony formation, cell migration, and apoptosis. (A) Over-expression efficiency of FBXW7 in HCT8 and Lovo cells was assayed by western blot. (B) FBXW7 over-expression inhibited the proliferation of HCT8 and Lovo cells. (C) The number of colony formation in CRC cells was reduced in the FBXW7 over-expression group compared to the control. (D) FBXW7 over-expression markedly inhibited HCT8 and Lovo cell migration. (E) The proportion of apoptosis in HCT8 cells was significantly increased in the FBXW7 over-expression group. All data were presented as the mean ± standard deviation and three independent experiments. * *p* < 0.05, ** *p* < 0.01, *** *p* < 0.001.

To clarify biological functions of FBXW7 in CRC, endogenous expression levels of FBXW7 in several CRC cell lines were measured by western blot assay. The different CRC cell lines showed different expression levels (**S1 Fig**). Among them, HCT-8 and Lovo CRC cells with low endogenous expression were used for transfecting FBXW7 over-expression plasmid. The efficiency of FBXW7 over-expression, as detected by western blot, was 4.98 and 5.3, respectively (**Fig 3A**). Functional analysis indicated that FBXW7 may decrease the proliferation, invasion, and migration ability of CRC cells (**Fig 3B–3D**). The induction of apoptosis in cells over-expressing FBXW7 was detected by Annexin V-FITC/PI double staining using flow cytometry; the data suggested that the number of apoptotic cells was significantly increased (**Fig 3E**), implying that FBXW7 also functions as a tumor suppressor gene in CRC.

### Pathway and metabolite alterations in CRC cells are affected by FBXW7 over-expression

**Fig 4:**
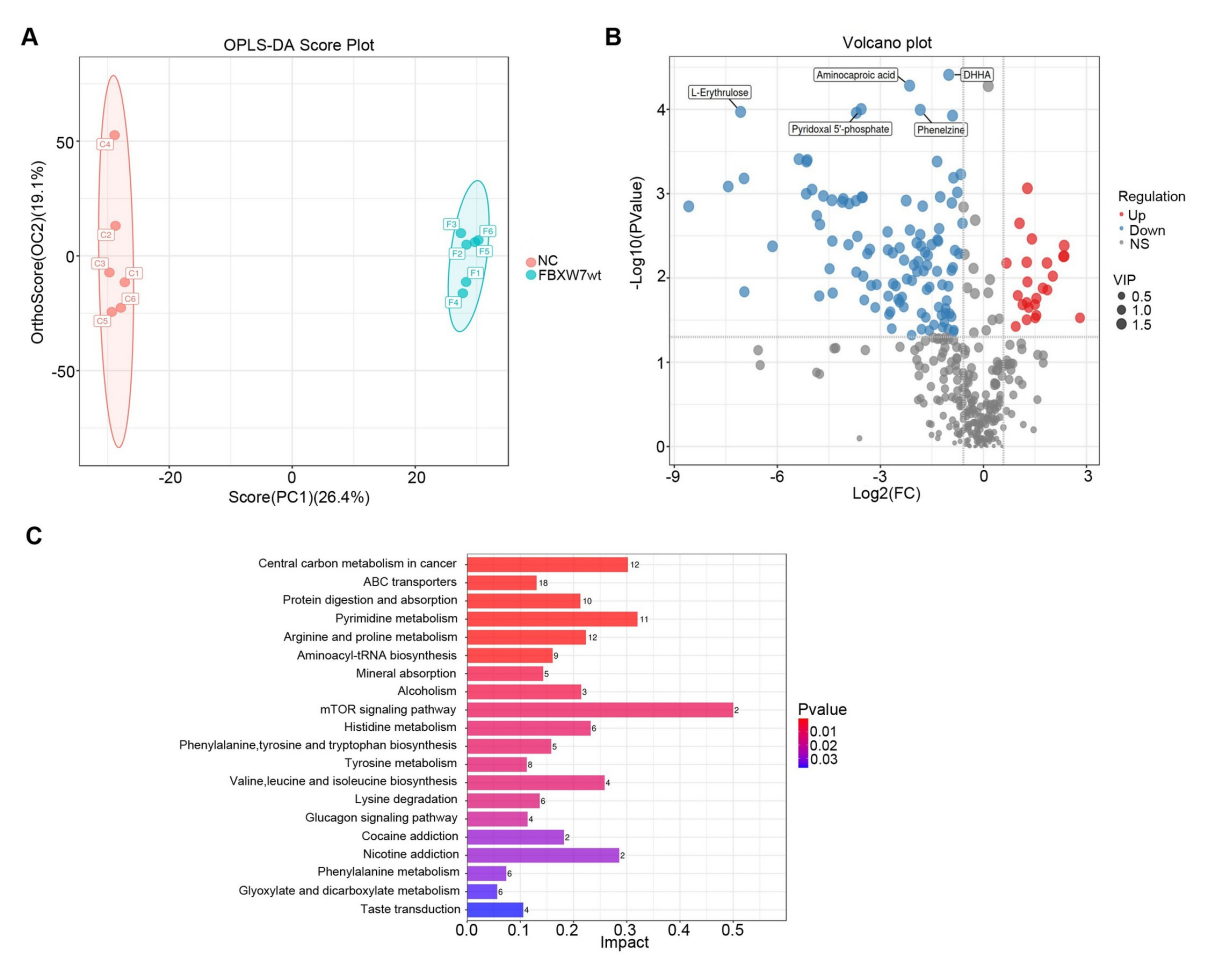
Key metabolic pathway components altered in FBXW7 over-expression cells. (A) An OPLS-DA score plot was created using the dataset of LC-MS. (B) Volcano plot of differential metabolites. (C) Pathway-enrichment analysis of differential metabolites.

Non-targeted metabolomic analysis was performed on FBXW7-overexpressing Lovo and control cells to detect distinct metabolites and expound the molecular mechanism of action of FBXW7 in CRC. In positive-ion mode, 1075 differential metabolites were identified, whereas 2934 differential metabolites were found in negative-ion mode. The principal component analysis (PCA) was performed on the dataset. The PCA score plot revealed a pattern of group separation. In the score plot, QC samples were grouped in a compact space, indicative of satisfactory stability and repeatability of the system (**S2 Fig**). An OPLS-DA score plot was created using this dataset. The FBXW7 over-expression group differed considerably from the control group and demonstrated adequate modeling and prediction abilities (*R*^2^_*X*_ = 0.541, *R*^2^_*Y*_ = 0.999, *Q*^2^_Cum_ = 0.787) (**Fig 4A**). The statistical test was carried out to determine the *p*-value, the OPLS-DA dimension reduction method was used to compute the variable significance in projection (VIP), and the fold change was utilized to calculate the multiple differences across groups. The strength and interpretability of the content of each metabolite component in the sample classification and discrimination were assessed, and metabolite screening was performed. The criteria for screening differential metabolites were VIP > 1, *p* < 0.05, and fc > 1.5. The first-level material list of samples revealed 1075 differential metabolites, of which 381 were up-regulated and 694 were down-regulated (**Fig 4B**). The metabolites were retrieved using the Human Metabolome Database (HMDB) (http://www.hmdb.ca), Massbank (http://www.massbank.jp/), LipidMaps (http://www.lipidmaps.org), mzCloud (https://www.mzcloud.org) confirmation annotation, and MS/MS fragment patterns. There were 133 distinct metabolites, of which 24 were up-regulated and 109 were down-regulated. The signaling pathways enriched by differential metabolites, such as the mToR signaling pathway, central carbon metabolism in cancer, pyrimidine metabolism, and nicotine addiction, indicate that FBXW7 may govern the incidence of CRC via these pathways (**Fig 4C**). Among these, the mTOR signaling pathway showed the highest effect value, indicating that the metabolites detected in this pathway contributed the most.

**Fig 5:**
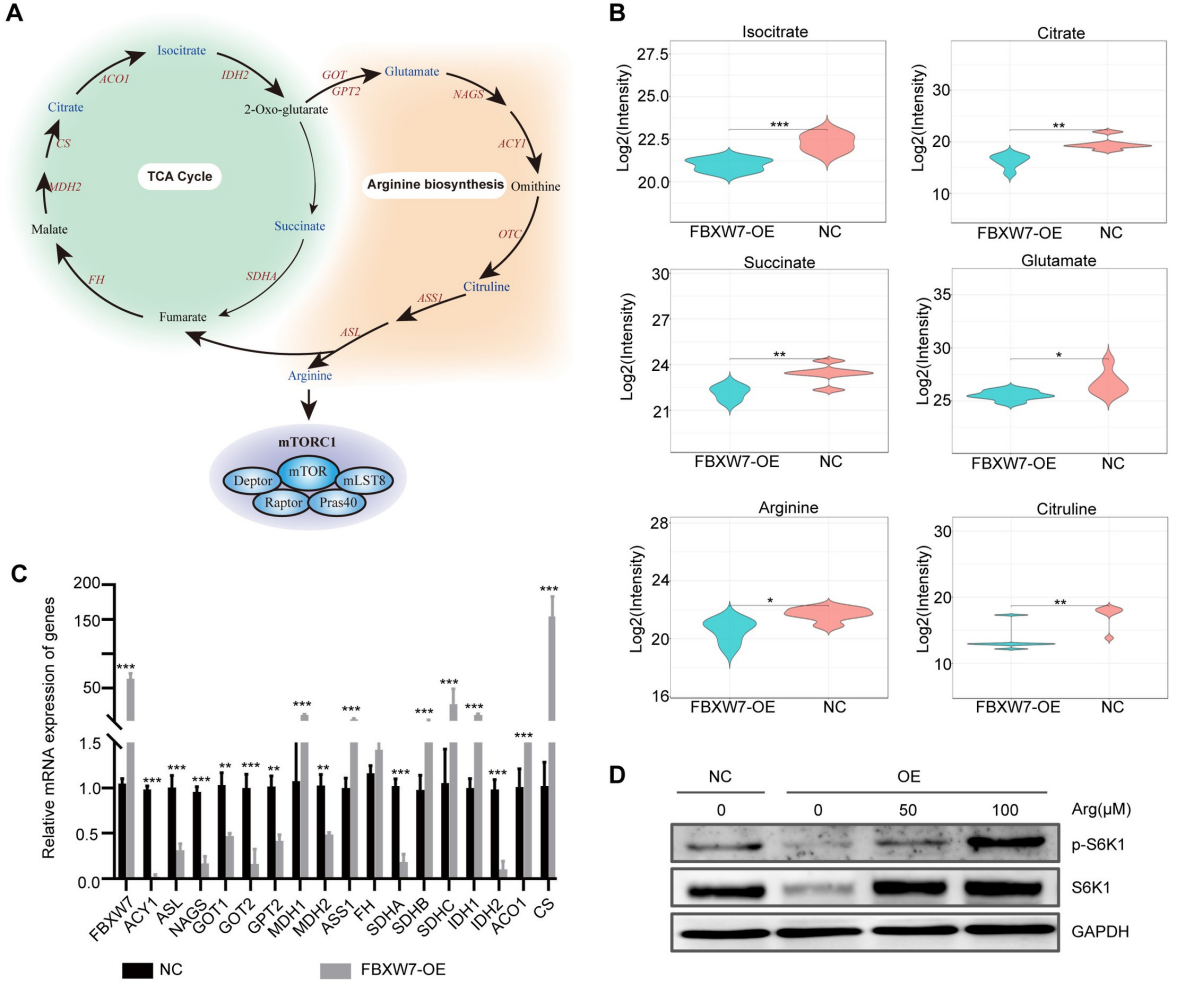
The change in the expression of mTOR pathway-related molecules. (A) mTOR signaling pathway-related metabolic pathways. (B) The metabolite changes of arginine biosynthesis and TCA cycle. (C) q-PCR was utilized to detect the mRNA level of metabolic enzymes in arginine biosynthesis and TCA cycle. (D) FBXW7 regulates mTORC1 by reducing the quantities of arginine. Statistical analysis was performed with a two-sided *t* test. * *p* < 0.05, ** *p* < 0.01, *** *p* <0.001.

Specifically, the FBXW7-OE group contained less L-arginine than the control group. Arginine has been shown to directly activate the mToR signaling pathway (**Fig 5A**). Therefore, the metabolites involved in arginine production were investigated. The quantities of isocitrate, citrate, citrulline, succinate, and glutamate associated with arginine synthesis decreased (**Fig 5B**). Furthermore, qPCR was utilized to assess the expression of relevant metabolic enzyme-coding genes in the cell types before and after FBXW7 over-expression. The results revealed that the expression levels of the arginine synthesis metabolic enzymes, aminoacylase-1 (ACY1), argininosuccinate lyase (ASL), glutamic-oxaloacetic transaminase 1 (GOT1), GOT2, isocitrate dehydrogenase 2 (IDH2), malate dehydrogenase 2 (MDH2), and succinate dehydrogenase complex flavoprotein subunit A (SDHA), were significantly down-regulated in FBXW7 over-expression cells (**Fig 5C**), indicating that the inhibitory effect of FBXW7 on arginine production may arise from changes in the expression of the associated metabolic enzymes. To test formally whether FBXW7 regulates mTORC1 by reducing the quantities of arginine, CRC cells engineered to over-express FBXW7 were leveraged. In contrast to normal cells, the abundance of phosphorylated (active form) forms of S6K1 in FBXW7-OE cells diminished, however, increasing extracellular concentrations of Arg from 0 to 100 μM in a dose-dependent manner enhanced the mTORC1 activity, as detected by the phosphorylation of its substrate S6K1 (**Fig 5D**).

### The expression of genes involved in arginine biosynthesis is negatively correlated with that of FBXW7

**Fig 6:**
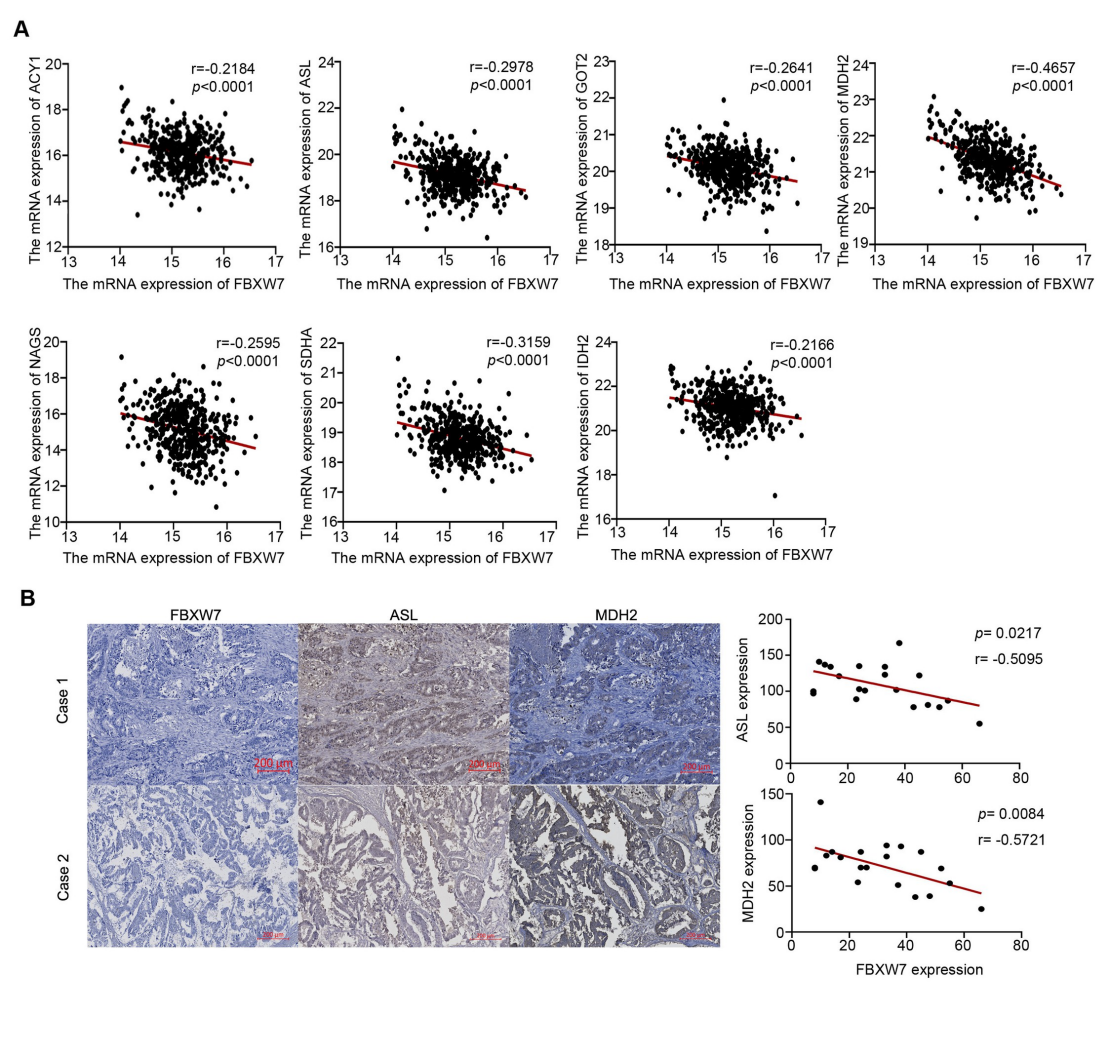
FBXW7 is negatively correlated with ASL and MDH2 in CRC. (A) FBXW7 mRNA was negatively correlated with ACY1, ASL, GOT2, MDH2, NAGS, SDHA, and IDH2 in 329 CRC samples based on TCGA data. (B) Immunohistochemistry was used to detect the expression of FBXW7, MDH2, and ASL in CRC samples. Statistical analysis was performed with Spearman correlation and a two-sided *t* test.

The relationship between FBXW7 and arginine-related metabolic enzyme-coding genes in CRC was explored using TCGA data. In the 329 CRC tissue samples, the expression of FBXW7 was negatively correlated with those of ACY1, ASL, ARG, IDH2, MDH2, SDHA, GOT1, GOT2, and GPT2 (**Fig 6A**). Furthermore, IHC tests were carried out on 20 CRC tissues; the findings revealed that the expression of FBXW7 was negatively correlated with that of ASL, MDH2, and IDH2 at the protein level (**Fig 6B**). These findings suggest that FBXW7 may down-regulate arginine production in CRC cells via metabolic reprogramming and limit CRC growth by decreasing the activation of mToR pathway.

## Discussion

The present study found that FBXW7 may have predictive significance in patients with CRC. Non-targeted metabolomics and *in vitro* tests revealed the anti-tumor activity of FBXW7 and that FBXW7 loss-of-function may lead to a malignant phenotype of CRC. To the best of our knowledge, the research findings show, for the first time, that FBXW7 may not only directly ubiquitinate the mTOR protein, leading to mTOR protein degradation, but also decrease arginine production via metabolic reprogramming, suppressing mTOR pathway activation and consequently the occurrence and progression of CRC.

The FBXW7 protein is a member of the F-box protein family and the target protein recognition component of the SCF E3 ubiquitin ligase complex [25]. FBXW7 is related to the survival rate of patients with cancer. Through a meta-analysis, Shang discovered that FBXW7 mutations or low expression might predict the OS rate of patients with CRC [27]. The expression of FBXW7 was shown to be lower in metastatic melanoma than in original melanoma and related to a poor 5-year survival rate [28]. Meta-analysis and bioinformatics unveiled that FBXW7 expression was associated with gastric cancer prognosis [29]. The expression of FBXW7 in CRC and matched normal tissues was initially examined using a tissue microarray and discovered that the FBXW7 protein was expressed in both the nucleus and cytoplasm. A low ratio of nuclear/cytoplasmic expression of FBXW7 was found to be related to poor outcome of individuals with CRC when combined with clinicopathological variables. Further univariate and multivariate COX regression model analyses suggested that low FBXW7 expression may be employed as an independent predictor of prognosis in CRC patients. The expression of FBXW7 mRNA and the clinicopathological factors were also examined using TCGA data, and the findings coincided with the results of the tissue microarray, confirming the potential prognostic value of low FBXW7 expression in patients with CRC.

Given that it is one of the major recognition factors in the ubiquitin-proteasome degradation pathway, many FBXW7 substrates are oncogenes. For example, cyclin E, c-Myc, c-Jun, and Notch are destroyed by the FBXW7-mediated ubiquitin-proteasome system [25,30,31]. FBXW7 mutations and deletions promote the accumulation of these oncogenes, which have been detected in ovarian, breast, and CRC tissues [32,33]. FBXW7 mutations or deletions are detected in approximately 9% of CRC cases [34]. Therefore, the role of FBXW7 in CRC cells was investigated. Over-expression studies were performed on two CRC cell lines with low endogenous FBXW7 expression. The results showed that FBXW7 over-expression suppressed the proliferation, invasion, and migration of CRC cells, demonstrating that FBXW7 functions as a tumor suppressor gene in CRC.

The mechanism by which FBXW7 functions as a tumor suppressor differs among cancer types. For example, in gastric cancer, FBXW7 causes apoptosis, arrests growth, and inhibits epithelial-mesenchymal transition by enhancing RhoA ubiquitination and proteasome degradation [35]. In colon cancer, FBXW7 inhibits enolase 1 activity via the ubiquitin/proteasome pathway in a glycogen synthase kinase 3-dependent manner [36]. FBXW7 suppresses tumor development and progression in ovarian cancer by degrading YTHDF2 and enhancing the stability of the pro-apoptotic gene Bcl-2-modifying factor [37]. By targeting MTDH degradation, FBXW7 can also suppress breast cancer growth and induce apoptosis [38]. By inhibiting FBXW7, miR-27a-3p enhances esophageal squamous cell carcinoma cell growth [39]. However, as a key E3 ubiquitinated protein ligase, the significance of FBXW7 in tumor metabolic reprogramming has been overlooked. Ubiquitination, the most prevalent post-translational protein modification, regulates cancer metabolic signaling pathways [19,21]. For instance, the deletion of von Hippel-Lindau Cullin RING E3 ligase can stabilize the expression of various glycolytic enzymes promoted by hypoxia-inducible factor 1-alpha in cancer cells to realize aerobic glycolysis. The E3 ligases HUWE1 and tumor necrosis factor receptor-associated factor 6 can ubiquitinate metabolic enzymes including hexokinase 2 and affect tumor growth by regulating glycolysis [40]. Therefore, understanding the role of FBXW7 in metabolic control is critical for analyzing CRC pathophysiology. Non-targeted metabolomics in CRC cells before and after FBXW7 over-expression could be detected and a series of differential metabolites were screened. After pathway enrichment analysis, the differential metabolites were found to show the most significant aggregation in the mTOR pathway. mTOR is a serine/threonine kinase that plays a role in tumor growth, angiogenesis, insulin resistance, fat creation, and other cellular processes, as well as in metabolism [41]. By boosting protein, lipid, and nucleotide synthesis and blocking catabolism, mTORC1 promotes glucose metabolism and accelerates cell growth and division [42]. The stability of the mToR also affects cytokine production in T cells and helps to regulate immunosuppression [43]. Therefore, mTOR has been identified as a novel target for tumor therapy. Rapamycin and its equivalents have been used to treat solid tumors, organ transplantation, rheumatoid arthritis, and other disorders [44]. The activation of the mTOR signaling pathway is controlled by four signals: growth factors, energy status, oxygen content, and amino acids [45]. Growth factors stimulate mTORC1 by activating the traditional insulin and Ras signaling pathways [41]. The energy status of the cell is then transmitted to mTORC1 via AMP-activated protein kinase [46]. Hypoxia exerts numerous effects on mTORC1 activity, including lowered ATP levels. Arginine is an important amino acid for mTORC1 activation and can well regulate mTORC1 signaling [47]. The metabolic studies unveiled that the amount of arginine in FBXW7-overexpressing CRC cells was dramatically reduced, which may explain why the mToR signaling pathway was inhibited.

Furthermore, metabolites associated with the arginine production pathway were discovered to be down-regulated. There are two major arginine biosynthesis pathways [48]: glutamic acid synthesis and arginine synthesis. Glutamine synthetase interacts with glutamic acid and ATP to generate glutamine and ADP, whereas arginine synthetase combines with glutamine and arginine to produce arginine and urea. In the alternative pathway, arginine transferase catalyzes the reaction between arginine and ATP to form arginine phosphate and AMP, which are then catalyzed by arginine synthase to produce arginine and ADP. L-citrulline is an important precursor for arginine production. Under the action of ACY, ornithine is transformed to citrulline. This is then transformed into argininosuccinic acid, which is subsequently converted to arginine and fumaric acid under the action of ASL. Fumaric acid is involved in the citrate cycle. Under the action of IDH2, α-ketoglutaric acid, a major intermediate metabolite of the tricarboxylic acid cycle, can be transformed into isocitrate/citrate and succinic acid. Citrulline, succinate (succinic acid), glutamate (glutamic acid), citrate (citric acid), and isocitrate levels were considerably lower in FBXW7-overexpressing CRC cells. Simultaneously, the expression of metabolic enzyme-encoding genes in metabolic pathways related to arginine synthesis was detected and the expression levels of nine metabolic enzyme-encoding genes (ACY1, ASL, IDH2, MDH2, SDHA, GOT1, GOT2, and GPT2) were down-regulated to varying degrees.

In addition, the aforementioned indicators in 20 pairs of human CRC tissues were detected to confirm the correlation between FBXW7 and arginine metabolism-related enzymes and found a negative correlation, as also confirmed by TCGA CRC data.

In summary, this research explored the relationship between the FBXW7 expression and clinicopathological variables in patients with CRC and explained its potential application value for prognosis. In the meantime, *in vitro* investigations revealed that FBXW7 may serve as a tumor suppressor gene in CRC, inhibiting proliferation and metastasis of CRC cells via two different mechanisms: reduction in arginine production through metabolic reprogramming and direct down-regulation of mToR signaling. The research findings provide a credible theoretical foundation for the prognostic prediction and therapeutic planning of CRC.

## Supporting information

S1 Fig The different expression level in different CRC cell lines detected by western blot. (TIF)

S2 Fig LC-MS was used to screen differential metabolites. (A)Differential metabolites in positive and negative ion modes. (B) Principal Component Analysis of Metabolites.
(TIF)

S1 Raw images Raw data and uncropped blots are depicted in the supplementary file. (PDF)

S1 Table Primers used in this study. (XLSX)

S1 Dataset (XLSX)
