# Supplementary figures and images for: FBXW7 metabolic reprogramming inhibits the development of colon cancer by down-regulating the activity of arginine/mToR pathways
Source: PLoS One. 2025 Jan 17;20(1):e0317294. doi: 10.1371/journal.pone.0317294 (PMC11741656; doi:10.1371/journal.pone.0317294)

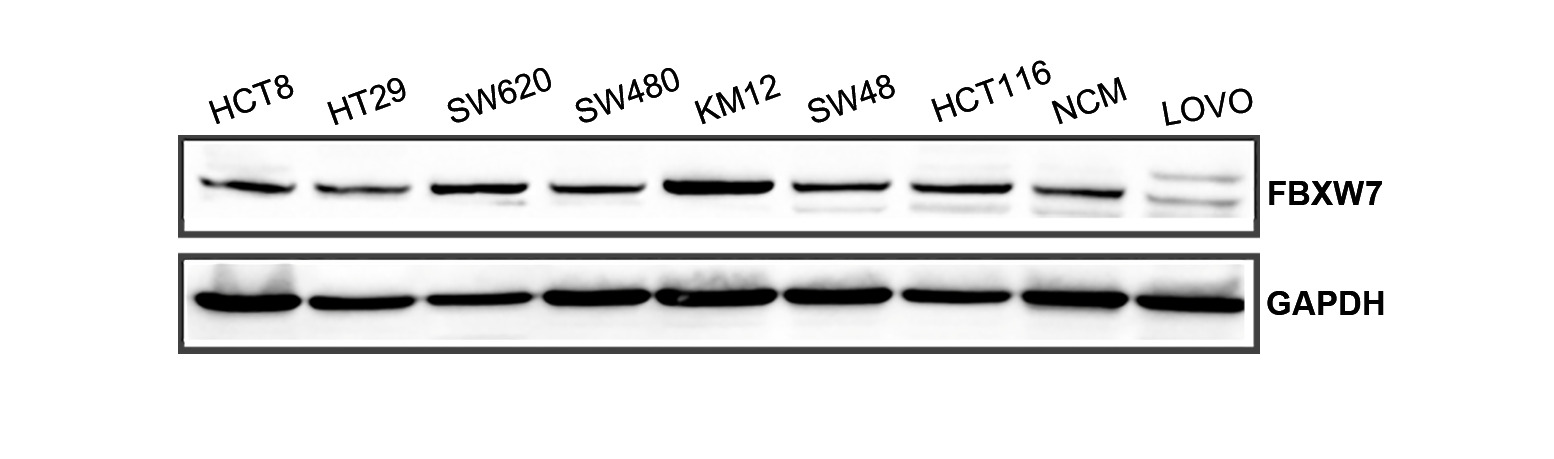

Supplement: S1 Fig — (TIF) [file pone.0317294.s001.tif]

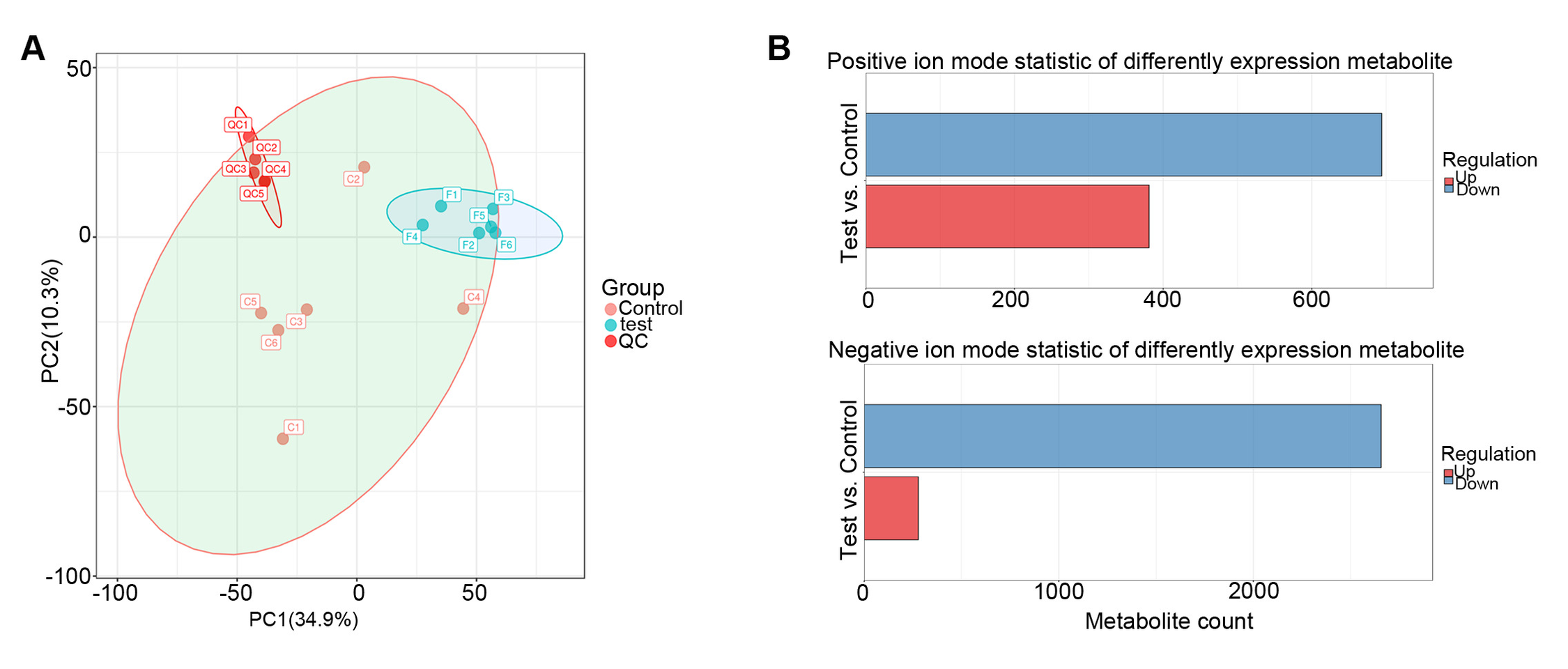

Supplement: S2 Fig — (A)Differential metabolites in positive and negative ion modes. (B) Principal Component Analysis of Metabolites. (TIF) [file pone.0317294.s002.tif]
