# Supplementary material for: FBXW7 metabolic reprogramming inhibits the development of colon cancer by down-regulating the activity of arginine/mToR pathways
Source: PLoS One. 2025 Jan 17;20(1):e0317294. doi: 10.1371/journal.pone.0317294 (PMC11741656; doi:10.1371/journal.pone.0317294)
Supplement: S1 Raw images — (PDF) [file pone.0317294.s003.pdf]

S1_ raw_data:
uncropped western blots

1. Images of raw data are organized according to their appearance in the manuscript or the supplementary.

2. Western blots were detected with the GelView 6000 Plus using 15s-20s increasing exposure times: Again images were exported using the software BioAnaly and the function “export raw data/TIF”. The most suitable exposure time (good signal-noise ratio and contrast) was selected.

Fig.5D

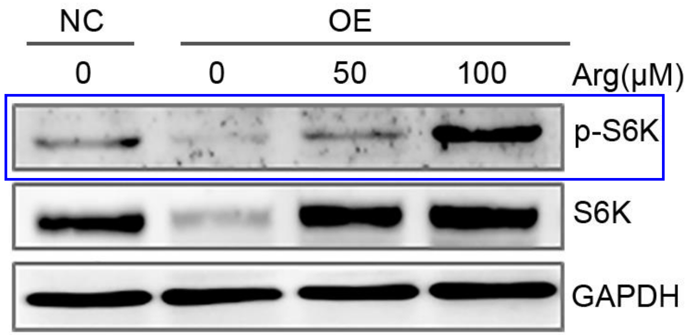

NC
OE
0
0
50
100
Arg(μM)
p-S6K
S6K
GAPDH

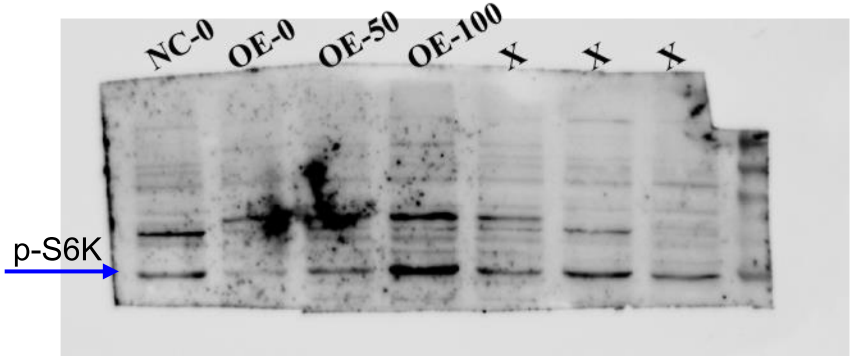

NC-0
OE-0
OE-50
OE-100
X
X
X
p-S6K

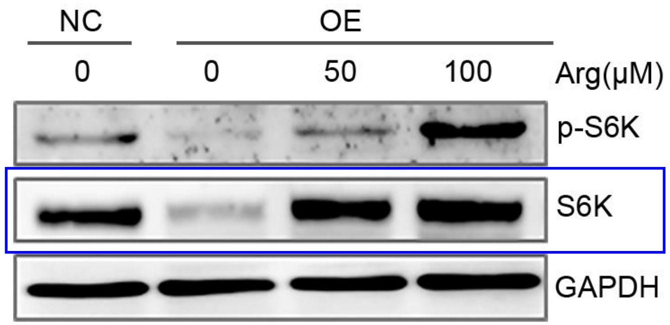

NC
OE
0
0
50
100
Arg(μM)
p-S6K
S6K
GAPDH

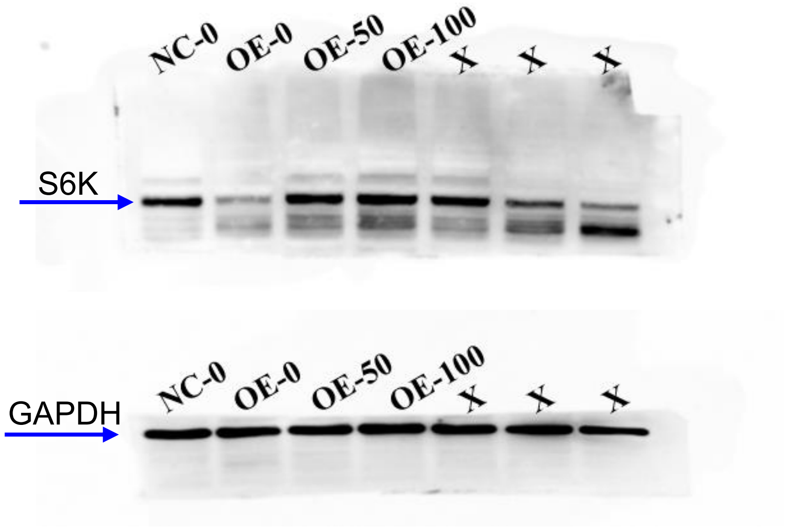

NC-0
OE-0
OE-50
OE-100
X
X
X
S6K
NC-0
OE-0
OE-50
OE-100
X
X
X
GAPDH

Fig.3A

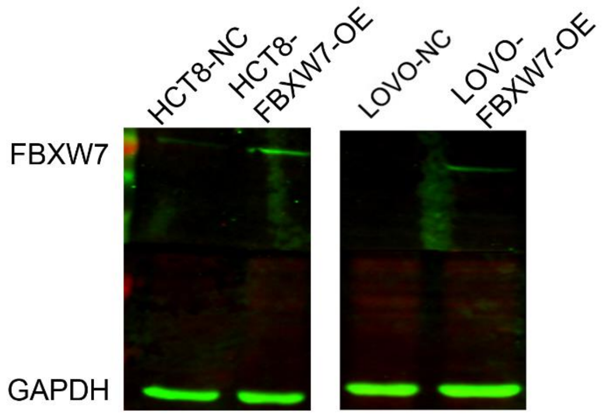

HCT8-NC
HCT8-FBXW7-OE
LOVO-NC
LOVO-FBXW7-OE
FBXW7
GAPDH

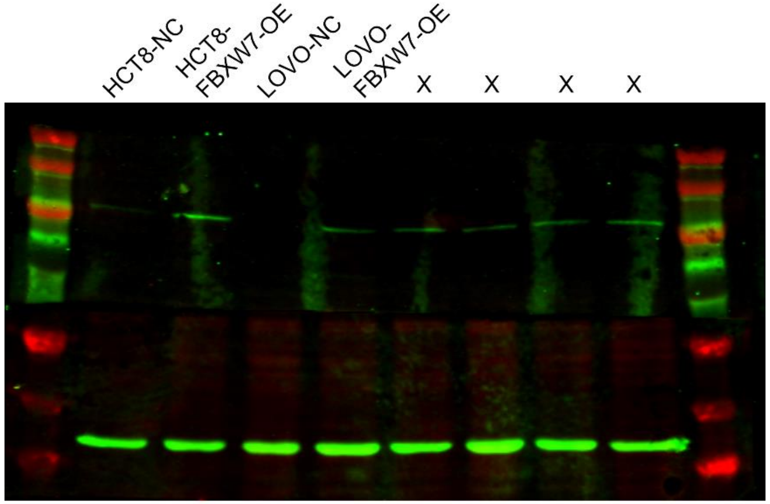

HCT8-NC
HCT8-FBXW7-OE
LOVO-NC
LOVO-FBXW7-OE
X
X
X
X

S1 Fig.

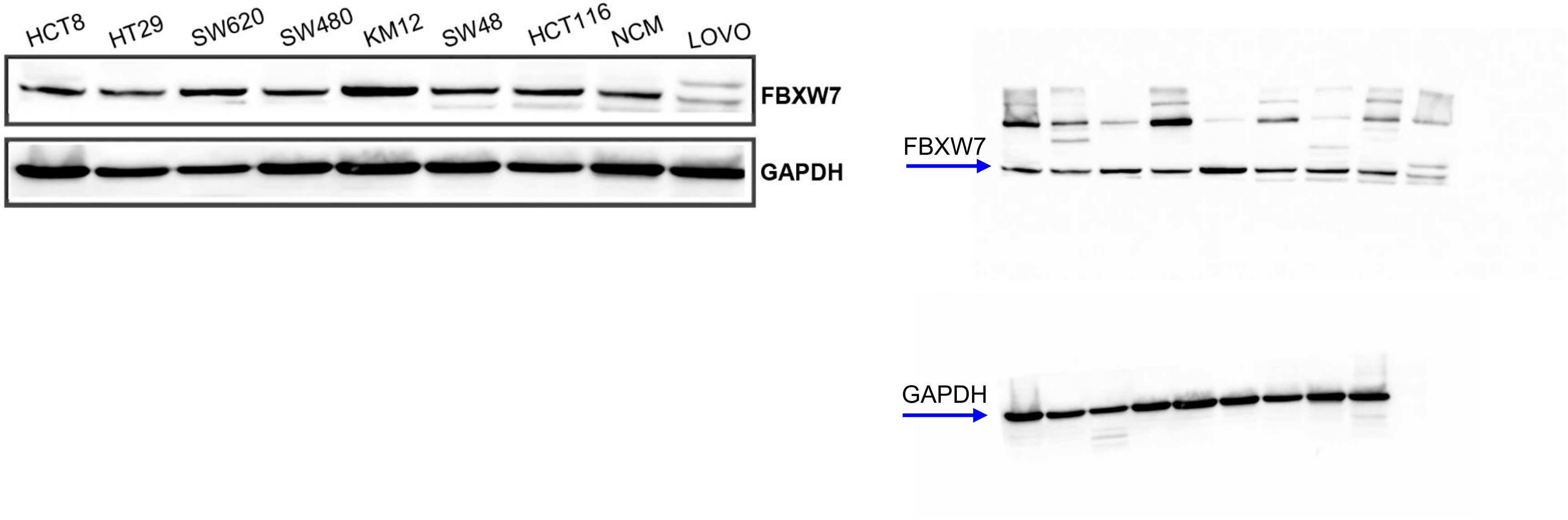
HCT8
HT29
SW620
SW480
KM12
SW48
HCT116
NCM
LOVO
FBXW7
GAPDH
FBXW7
GAPDH
